# Do Children and Adolescents with Overweight or Obesity Adhere to the National Food-Based Dietary Guidelines in Greece?

**DOI:** 10.3390/children9020256

**Published:** 2022-02-15

**Authors:** Alexandra Georgiou, Odysseas Androutsos, Giorgos Chouliaras, Evangelia Charmandari

**Affiliations:** 1 First Department of Paediatrics, School of Medicine, National and Kapodistrian University of Athens, “Aghia Sophia” Children’s Hospital, 11527 Athens, Greece; alsgeorgiou@gmail.com (A.G.); georgehouliaras@msn.com (G.C.); 2 Department of Nutrition and Dietetics, School of Physical Education, Sport Science and Dietetics, University of Thessaly, 42132 Trikala, Greece; oandroutsos@uth.gr; 3 Division of Endocrinology and Metabolism, Center of Clinical, Experimental Surgery and Translational Research, Biomedical Research Foundation of the Academy of Athens, 11527 Athens, Greece

**Keywords:** obesity, nutrition, national guidelines, adherence

## Abstract

Childhood obesity increases the risk for metabolic disorders, but is also related to nutritional deficiencies, such as anemia and hypovitaminosis D. Although children/adolescents with overweight/obesity may have higher energy intake, their diet quality and diversity may be low. The present study aimed to evaluate the consumption of foods against the national food-based dietary guidelines in children and adolescents with overweight or obesity in Greece. Sociodemographic, anthropometric and lifestyle data were collected from a sample of 1467 children 2–18 years old (962 obese, 505 overweight, 51.2% females) in 2014–2017. The results of this study show that the consumption of dairy products, fruit, vegetables, legumes and fish by children/adolescents with overweight or obesity was lower than the national recommendations (ranging from a minimum of 39.5% for fish, to a maximum of 75.5% for cereal/potato/rice). Only the consumption of meat/poultry was found to exceed the national recommendation (estimated coverage of 131.3%). Moreover, a large proportion of participants regularly consumed various unhealthy foods/beverages. The present findings indicate that the majority of children/adolescents with overweight/obesity do not comply with the national food-based dietary guidelines in Greece. The implementation of new strategies to promote healthy diets among children/adolescents with overweight/obesity are urgently required.

## 1. Introduction

Childhood obesity has significantly increased over the past decades [1]. Previous studies have shown that the prevalence of overweight and obesity among children and adolescents in Greece is high compared with other European countries. More specifically, the ToyBox study revealed that 20.6% of preschool children in Greece were overweight/obese, while the Healthy Growth Study and the ENERGY project showed that this figure almost doubles at the age of 10–12 [2,3,4].

The etiology of obesity is complex and attributed to a wide range of genetic, perinatal, socioeconomic and lifestyle factors that have been associated with this disease [5,6]. It may have detrimental effects on health, with significant short-term implications for children, including metabolic abnormalities, musculoskeletal and breathing problems, and psychosocial effects [1]. Furthermore, individuals with overweight or obesity in childhood are at higher risk of being overweight or obese in adulthood and, consequently, at higher risk for the development of non-communicable diseases, such as type 2 diabetes mellitus, hypertension and certain types of cancer [7,8,9]. Beyond its health consequences, obesity also causes financial burden to the national health systems due to the high expenses required for the management of its complications [10].

It is suggested that the main cause of obesity is attributed to an energy imbalance maintained for long periods, resulting from a higher energy intake compared with energy expenditure [1]. Furthermore, previous studies have highlighted the so-called “double burden of disease”, according to which childhood obesity is not only associated with metabolic abnormalities, but also with the development of certain nutritional deficiencies, such as vitamin D insufficiency or deficiency, and anemia [11]. It might be speculated that high energy intake and poor diet quality determine these health conditions in childhood obesity. However, the findings from the National Health and Nutrition Examination Survey (NHANES) (2009–2014) showed that diet quality does not significantly differ between children with overweight/obesity and their normal-weight peers [12]. Similarly, the GRECO study in Greece showed that adherence to a Mediterranean diet, as assessed with valid indices, such as the KIDMED, did not differ significantly between normal-weight and overweight or obese schoolchildren [13]. The findings regarding potential differences in caloric intake between children with overweight or obesity and their normal-weight peers are contradictory, possibly due to methodological barriers (e.g., self-reported data; targeting the general population, thus having a small number of children with overweight/obesity; etc.) [14,15].

To better understand the determinants of childhood obesity and assess children’s nutrition, previous studies have also explored children’s adherence to food-based dietary guidelines. According to the findings of two European studies, the EsKiMo II study, which included a nationwide, representative sample of 1353 volunteers aged 12–17 years in Germany, and the HELENA study, which included 1593 volunteers aged 12.5–17.5 years from ten European cities, adolescents’ consumption of several core foods (e.g., fruit, vegetables, milk/dairy products) was below the recommendations, whereas the consumption of meat/meat products exceeded the recommendations [16,17]. The ToyBox study, which included 3301 preschool children from six European countries, showed that about 35% and 55% of children with normal weight did not comply with the recommendations on fruit and vegetables consumption, respectively [18]. In Greece, the national food-based dietary guidelines were developed and became available in 2014. According to these guidelines, children and adolescents are advised to consume foods from all food groups (i.e., fruit, vegetables, milk and dairy products, bread, cereals and potatoes, legumes, meat, egg, fish and seafood, nuts and oils) on a daily or weekly basis, with the recommended servings varying according to children’s age and gender. Still, no previous study has examined if children and adolescents with overweight/obesity in Greece comply to these guidelines.

The aim of the present study was to explore if children and adolescents with overweight or obesity in Greece adhere to the national food-based dietary guidelines, using a large-scale cohort consisting only of children/adolescents with overweight/obesity.

## 2. Materials and Methods

### 2.1. Study Design and Participants

The present study was a cross-sectional study. Children and adolescents aged 2–18 years attending the out-patient clinic for the prevention and management of overweight and obesity in childhood and adolescence were recruited between October 2014 and March 2017. The following inclusion criteria were applied: living in Greece, being able to complete the study questionnaire in Greek language, child/adolescent aged 2–18 years with overweight/obesity and providing a consent form. For all age groups of children and adolescents, the parents were initially approached and informed about the study purpose and protocol, and they were asked to provide a written consent to enroll their child to the study. This common recruitment approach was followed since all children/adolescents were brought to the clinic by their parents. Additionally, all children and adolescents gave their verbal assent to proceed with the measurements.

The study adhered to the Declaration of Helsinki and the conventions of the Council of Europe on human rights and biomedicine. The study was approved by the Committee on the Ethics of Human Research of ‘Aghia Sophia’ Children’s Hospital (Approval Number: EB-PASCH-MoM: 28 November 2013, Re: 10290-14 May 2013). Written informed consent was obtained in all cases by a parent, and assent was given by children older than 7 years. Further data regarding the whole study design can be found in detail elsewhere [19]. Children and adolescents were classified as “overweight” or “obese” according to the International Obesity Task Force (IOTF) cut-off points.

### 2.2. Procedure

All participants were admitted to the Endocrine Unit early in the morning on the day of the assessment, and a detailed medical history and clinical examination, including standard anthropometric measurements (weight, height, waist circumference, hip circumference) were obtained by one trained researcher. A complete interview with the accompanying parents/caregivers was performed regarding demographic and socioeconomic data, and data regarding the family composition (number of children, married/divorced parents, the participant’s line of birth between the family’s offspring) and children’s/adolescents’ consumption of foods.

### 2.3. Instruments and Variables

Data regarding children’s/adolescents’ usual food and beverage habits over the last year before the assessment were collected from the parents/caregivers by completing a food frequency questionnaire (FFQ), which has been validated in Greek subjects [20,21,22]. In brief, this FFQ comprised questions regarding the frequency of consumption of thirty-seven food and beverage items. Response categories were: “never or less than once per month”, “1–3 days per month”, “1 day per week”, “2–4 days per week”, “5–6 days per week” and “every day”. Next, the average consumption of each food or beverage category per day was asked. Parents/caregivers were asked to indicate the portion size category that best fit the daily portion of their child, between specific response categories depending on the food item. A list of common standard measures was given as examples for each food or beverage category. The average daily intake for each food and beverage category was estimated by multiplying the number of days per week with the average consumption per day and then dividing the result by 7. The portions per day that were consumed by each child/adolescent were calculated by dividing the result obtained in the previous step by the portion size, which is described for children and adolescents in the Greek food-based national recommendations. Children who had no data on both frequency and portion size for all thirty-seven items were excluded from the study. The frequency of consumption of the three main meals (breakfast, lunch and dinner) was assessed based on three relevant questions asking how many days per week children eat each main meal, with the responses varying in a six-point scale ranging from “(almost) never” to “every day”. The FFQ also included four questions regarding the frequency at which the child consumes a food or beverage between the main meals (with a response range between “never or less than once per month” to “every day”), and what kind of food and/or beverage they usually choose.

The Greek national food-based guidelines were used to compare participants’ food consumption. From the list of 37 food items included in the FFQ, all kinds of fruit (fresh fruit, dried fruit, canned fruit and freshly squeezed/home-made fruit juice) were grouped into one category (i.e., “fruit”). Similarly, all kinds of vegetables (i.e., fresh vegetables and cooked vegetables) were grouped into one category (i.e., “vegetables”). Plain milk, plain yoghurt, fruit/sugared/aromatized yoghurt and cheese were grouped into one category (“dairy”). Unsweetened breakfast cereals, sweetened breakfast cereals, white bread and other bakery products, brown or wholemeal bread and other bakery products, pasta, rice, deep-fried potato products and potatoes were grouped into one category (“cereal/potato/rice”). Legume consumption corresponds to the category “legumes”. The portion that was consumed by each child/adolescent per day was compared with the age-specific recommended portion, which is described in the Greek food-based national guidelines for children and adolescents, leading to three categories of consumers: “according to recommendation”, “less than recommended” and “more than recommended”.

The food items which could not be matched to the food groups of the national food guidelines are presented as single food items. No data were recorded regarding added lipids/oils/nuts or eggs, so, for these two food groups, no comparisons against the national food recommendations were possible.

### 2.4. Statistical Analysis

Continuous data are presented as mean ± standard deviation, and median (interquartile range), whereas categorical variables are presented as absolute and relative frequencies. Pie charts are used to present graphically categorical outcomes. Student’s t-test was used for comparison of continuous data between two groups and one-way analysis of variance (ANOVA) with Bonferroni corrections for post hoc t-tests implemented for comparisons between more than two groups. Comparisons of percentage of recommended consumption and absolute deviation from recommended consumption were performed between obese and overweight children, males and females, and age class (2–6, 6–12 and 12–18 years). All statistical analyses were performed in Stata 11.2 statistical software (StataCorp, College Station, TX, USA).

## 3. Results

A total of 1540 children were recruited and 1467 (962 obese, 505 overweight, 51.2% females) with a mean age of 10.4 ± 3.0 years (range: 2–18) were included in the analysis. Descriptive demographic and socioeconomic data are summarized in Table 1. Approximately 25% (394 children) had been, previously, subjected to some type of intervention, aiming at reducing body weight. The most frequent was dietary counseling (88.3%), whereas other reported interventions included psychological support (6.4%), physical activity programs (1.7%) and a combination of interventions (3.6%). In more than half of the participating children (54.4%), both parents were classified as overweight/obese (BMI > 25 Kg/m^2^), whereas in 37.1%, only one parent was overweight/obese. Only 8.5% of the included children had both parents with BMI < 25 Kg/m^2^.

Table 2 illustrates the average consumption of food groups presented as absolute values, percentage of recommended consumption and absolute deviation from recommended consumption. It can be seen that for all food groups, with the striking exception of meat/poultry, the consumption was lower than the national recommendations. The average coverage ranged from a minimum of 39.5% for fish, to a maximum of 75.5% for cereal/potato/rice. Meat/poultry was the only food group that, on average, was consumed in excess of the national recommendations, with an estimated coverage of 131.3%.

**Figure 1:**
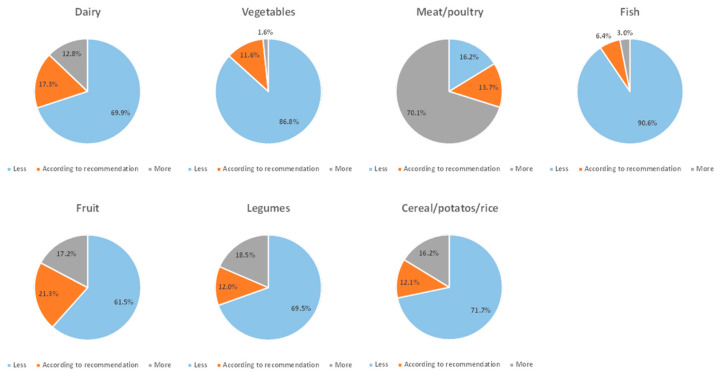
Compliance (less, as suggested or more) of the average consumed quantity of food groups to national recommendations.

The classification of the participants according to whether the consumed quantities followed the national recommendations (i.e., less, as suggested or more) is presented in Figure 1.

The frequency of consumption of non-recommended food groups is shown in Table 3.

## 4. Discussion

The present study is the first study in Greece that describes the compliance of children with overweight/obesity with the national food-based guidelines (Table S1). The findings of this study reveal that the majority of participating children/adolescents with overweight/obesity consume less quantities of food groups than those proposed in the Greek food-based recommendations for these age groups, except for meat/poultry, which is over-consumed. Additionally, participants were found to frequently consume unhealthy foods and beverages. It is noted that two of the food categories which are included in the Greek national food-based guidelines (i.e., added lipids/oils/nuts and eggs) were not recorded in the present study. Therefore, no conclusions can be reached regarding the compliance of the study sample against these specific food categories.

Low diet quality may lead to significant health problems in youth. Childhood and adolescence comprise two main periods of growth and the dietary requirements of energy, macro- and micro-nutrients are high during this stage of life. Inadequate consumption of core foods and/or overconsumption of unhealthy foods may lead to malnutrition, metabolic abnormalities and insufficiency or deficiency of micronutrients, such as minerals and vitamins. Moreover, the adoption of unhealthy dietary patterns in these age groups increases the likelihood of retaining them in the next stages of life and, consequently, the risk for developing specific non-communicable diseases [1,7,8,9].

The findings of the present study are in line with those of the study by Brettschneider et al., which showed that the majority of adolescents (all weight categories) did not comply with the German national dietary guidelines [16]. Specifically, their consumption of fruit, vegetables, milk/dairy products and starchy foods was below the recommended quantity, but their consumption of meat/meat products exceeded the recommendations. Similarly, the HELENA study showed that European adolescents did not meet the food-based guidelines “Optimized Mixed Diet” and “Food Guide Pyramid”, since they consumed a lesser quantity of fruit, vegetables and milk/milk products, and a greater quantity of meat/meat products [17]. In younger age groups, the findings of previous studies are also similar to those of the current study. In a sample of 521 children aged 5–10 years (all weight categories), Kunin-Batson et al. showed that only 14% of children met the guideline of five servings of fruit/vegetables per day, and 42% met the guidelines regarding the consumption of sugar-sweetened beverages, while the percentage of those meeting both guidelines was even lower (8%) [23]. Still, no differences between overweight/obese children and their normal weight peers were identified. The ToyBox study, which focused on a large number of European preschool children from six European countries, and used the same FFQ as the one used in the present study, showed that no clear differences were observed in diet quality assessed by the DQI index between overweight/obese and normal-weight children [24]. However, based on the same study sample, Cardon et al. showed that the mean consumption of soft drinks and fruit was significantly higher in overweight/obese preschool children compared with their normal-weight peers [18]. Similarly, the present study showed that >37% of the participants consumed soft drinks (with added sugar) at least once per week. Further studies are needed to confirm if the differences between children with normal weight and overweight/obesity are focused only on specific foods, and to elucidate if the overconsumption of these foods may impact overweight/obese children’s nutritional status. Future initiatives should aim to improve the adherence of children/adolescents with overweight/obesity to the food-based guidelines and lower their consumption of unhealthy foods. In this direction, a series of multilevel actions is required. A recent study in Greek schoolchildren aged 10–12 years and two systematic reviews suggested that low health literacy is associated with unhealthy dietary behavior (e.g., less frequent consumption of breakfast, healthy snacks, main meals and family meals) and childhood obesity [25,26,27]. Therefore, it is important to increase health literacy in the general population, to raise their awareness on the detrimental effects of unhealthy nutrition in childhood and adulthood. Considering that low socioeconomic groups may have lower health literacy, these vulnerable subgroups of the population should be prioritized and targeted [28]. Furthermore, actions to promote healthy eating and avoid unhealthy food consumption should be taken. These should include practices related to the improvement of the school food environment and the implementation of new communication strategies to reach children/adolescents. The study of Lien et al. showed that only 46% of the primary schools in Greece that participated in the ENERGY project prohibited children from bringing unhealthy foods/drinks to school [29]. This highlights the necessity of adopting policies and programs to promote healthy eating at school, which, according to FAO, is an essential element to foster healthy diets at school and has been previously shown to combat unhealthy food consumption [30,31]. In addition, previous studies suggested that healthy diets could be promoted through communication channels that are appealing to children and adolescents (e.g., social media and gamification), in order to reach broader population groups, and especially those who live with overweight and obesity [32]. For example, a recent systematic literature review and meta-analysis by Suleiman-Martos showed that gamification could be an effective method to improve knowledge of healthier nutritional habits in children and adolescents [32]. Moreover, the systematic literature review by Chau et al. suggested that social media is a promising feature for nutrition interventions in adolescents [33].

The findings of the current study should be interpreted under the light of its strengths and limitations. Regarding its strengths, the study included a large sample size, while all study procedures were harmonized, following specific protocols, validated questionnaires and equipment. Additionally, all measurements were taken by trained personnel and FFQs were completed during interviews with the parents. On the other hand, the study limitations include the study sample, which consists only of children with overweight/obesity, as well as the cross-sectional design of the study, which does not allow the establishment of causal relationships between adherence to the dietary guidelines and risk for overweight/obesity. Moreover, the study sample is not representative. It should also be mentioned that dietary data were self-reported, which may be subject to recall bias and socially desirable answers. Another limitation of the present study is that data regarding children’s/adolescents’ consumption of eggs and of added lipids/oils/nuts was not recorded; therefore, it was not feasible to report the degree of their compliance against the national food recommendations. Future studies are needed to elucidate if Greek children/adolescents with overweight or obesity comply with the national food recommendations for added lipids/oils/nuts and eggs consumption.

## 5. Conclusions

The present study showed that a large number of the participating children and adolescents with overweight or obesity did not comply with the national food-based dietary guidelines. Considering the detrimental effect of poor diet quality on health, urgent actions are needed to promote healthy nutrition and treat overweight and obesity in childhood and adolescence.

## Figures and Tables

**Table 1:** Socioeconomic and demographic data.

Parameter	N	%
Origin		
Greek	1358	92.6%
Other	109	7.4%
Residency		
Major urban center	1294	88.2%
Rural areas	173	11.8%
Single-parent family, yes	224	15.3%
Stay with relatives, yes	362	24.7%
Systemic exercise, yes	839	57.2%
Sports at school, yes	1277	87.1%
Frequency of physical activity (days per week)		
1–2	459	31.3%
3	565	38.5%
>3	443	30.2%
Smoking, yes	11	0.77%
Alcohol, yes	8	0.56%
Drugs, yes	0	0%

**Table 2:** Average consumption of food groups expressed as absolute values of consumption, percentage of recommended consumption and absolute deviation from recommended consumption.

	Average Consumption	Average Consumption as Percentage (%) of Recommendation	Average Absolute Deviation from Recommended Consumption
Dairy (daily servings)	2.1 ± 1.1, 2.0 (1.4, 2.7)	73.2 ± 32.5, 74.3 (49.7, 100.0)	−0.7 ± 1.0, −0.7 (−1.4, 0)
Vegetables (daily servings)	0.8 ± 0.5, 0.7 (0.3, 1.1)	45.3 ± 31.2, 38.9 (19.3, 68.6)	−1.1 ± 0.8, −1.0 (−1.6, −0.5)
Meat/poultry (weekly servings)	3.8 ± 1.8, 3.8 (2.9, 5.1)	131.3 ± 54.2, 127.7 (100.0, 170.8)	1.0 ± 1.5, 0.8 (0, 2.1)
Fish (weekly servings)	0.9 ± 0.8, 0.7 (0.4, 0.9)	39.5 ± 30.1, 36.5 (20.1, 47.9)	−1.2 ± 0.6, −1.3 (−1.6, −1.0)
Fruit (daily servings)	1.5 ± 1.1, 1.3 (0.8, 2.1)	75.0 ± 45.9, 75.9 (44.1, 100.0)	−0.5 ± 0.9, −0.4 (−1.2, 0)
Legumes (weekly servings)	1.7 ± 1.5, 1.1 (0.8, 2.0)	52.7 ± 40.3, 40.0 (25.0, 75.0)	−1.3 ± 1.2, −1.6 (−2.2, −0.7)
Cereal/potato/rice (daily servings)	3.9 ± 2.1, 3.5 (2.4, 5.0)	75.5 ± 35.4, 71.8 (49.5, 100.0)	−1.2 ± 1.9, −1.3, (−2.3, 0)

Data are presented as mean ± standard deviation, median (interquartile range) of daily or weekly servings.

**Table 3:** Frequency of consumption of non-recommended food groups.

	Never or Less than 1 per Month	1–3 Times per Month	1 Time per Week	2–4 Times per Week	5–6 Times per Week	Everyday
Sugared milk	75.5%	11.6%	4.2%	3.6%	1.3%	3.8%
Soft drinks	38.1%	24.7%	18.5%	13.3%	2.4%	3.0%
Light soft drinks	67.1%	13.5%	8.8%	7.5%	1.0%	2.1%
Bottled fruit juice	23.3%	23.3%	18.4%	24.7%	5.0%	5.3%
Tea	68.7%	15.3%	6.4%	7.1%	1.1%	1.4%
Smoothies	82.5%	8.3%	4.3%	3.7%	0.4%	0.8%
Chocolate	9.3%	19.6%	25.0%	31.5%	7.5%	7.1%
Chocolate spread	23.8%	22.2%	19.9%	24.5%	4.5%	5.1%
Milk-based desserts	77.3%	10.5%	5.4%	4.6%	0.6%	1.6%
Cakes	18.0%	36.1%	21.7%	19.2%	2.5%	2.5%
Biscuits	14.1%	25.9%	22.7%	26.4%	5.7%	5.2%
Pastries	30.4%	28.1%	20.1%	15.9%	2.1%	3.4%
Sugar-based desserts	41.9%	22.6%	12.4%	15.1%	3.8%	4.2%
Salty snacks	36.2%	30.3%	18.9%	11.4%	1.9%	1.3%
Processed meat products	14.3%	8.1%	8.9%	33.4%	14.1%	21.2%

## Data Availability

The data presented in this study are available on request from the corresponding author. The data are not publicly available due to data protection issues.

## Supplementary Materials

The following are available online at https://www.mdpi.com/article/10.3390/children9020256/s1, Table S1: Recommended dietary intake according to the Greek national guidelines for children and adolescents.
