# Supplementary material for: Do Children and Adolescents with Overweight or Obesity Adhere to the National Food-Based Dietary Guidelines in Greece?
Source: Children (Basel). 2022 Feb 15;9(2):256. doi: 10.3390/children9020256 (PMC8870155; doi:10.3390/children9020256)
Supplement: Supplementary file 1 [file children-09-00256-s001.zip › children-1535790-supplementary.pdf]

Table S1. Recommended dietary intake according to the Greek National guidelines for children and adolescents (www.diatrofikoiodigoi.gr)

Food group	Age groups				Portion examples
	2-3 years	4-8 years	9-13 years	14-18 years	
Vegetables	1 portion/day	1-2 portions/day	2-3 portions/day	3-4 portions/day	150-200gr of fresh or cooked vegetables
Fruits	1 portion/day	1-2 portions/day	2-3 portions/day	3 portions/day	120-200gr of fruit
Milk & dairy products	2 portions/day	2-3 portions/day	3-4 portions/day	3-4 portions/day	1 cup (240ml) of milk, 1 cup (200gr) of yogurt, 30gr of hard cheese, 2 tbs of soft cheese, 1 slice of cheese, ½ cup (125ml) of condensed milk
Cereals	3 portions/day	4-5 portions/day	5-6 portions/day	6-8 portions/day	1 slice (30gr) of bread, 1 medium rusk, ½ cup (70-90gr) of cooked pasta or rice or bulgur, ½ cup (30gr) of breakfast cereals, 1 medium potato (120-150gr cooked)
Lentils	up to 3 portions/week	3 portions/week	at least 3 portions/week	at least 3 portions/week	-
	60-90gr per portion	90-120gr per portion	120-150gr per portion	150-200gr per portion	
Red and white meat	2-3 portions/week	2-3 portions/week	2-3 portions/week	2-3 portions/week	(grams of portions refer to boneless cooked meat)
	60gr per portion	60-90gr per portion	90-120gr per portion	120-150gr per portion	
Eggs	4-7 per week				-

Fish and seafood	2 portions/week<br>60-90gr per portion	2-3 portions/week<br>90-120gr per portion	2-3 portions/week<br>120-150gr per portion	2-3 portions/week<br>150gr per portion	(grams of portions refer to boneless cooked fish)
Added fats and oils, olives and nuts	1-2 portions/day	2-3 portions/day	3-4 portions/day	4-5 portions/day	1 tbs of olive oil or other vegetable oil, 1 handful of nuts (18 almonds, 6 whole walnuts, 3 tbs of sunflower seeds), 10-12 olives, 1,5 tbs (25gr) of tahini, 1 tbs (15ml) butter or margarine
